# Supplementary figures and images for: Mechanisms of action and in vivo antibacterial efficacy assessment of five novel hybrid peptides derived from Indolicidin and Ranalexin against Streptococcus pneumoniae
Source: PeerJ. 2017 Oct 5;5:e3887. doi: 10.7717/peerj.3887 (PMC5632533; doi:10.7717/peerj.3887)

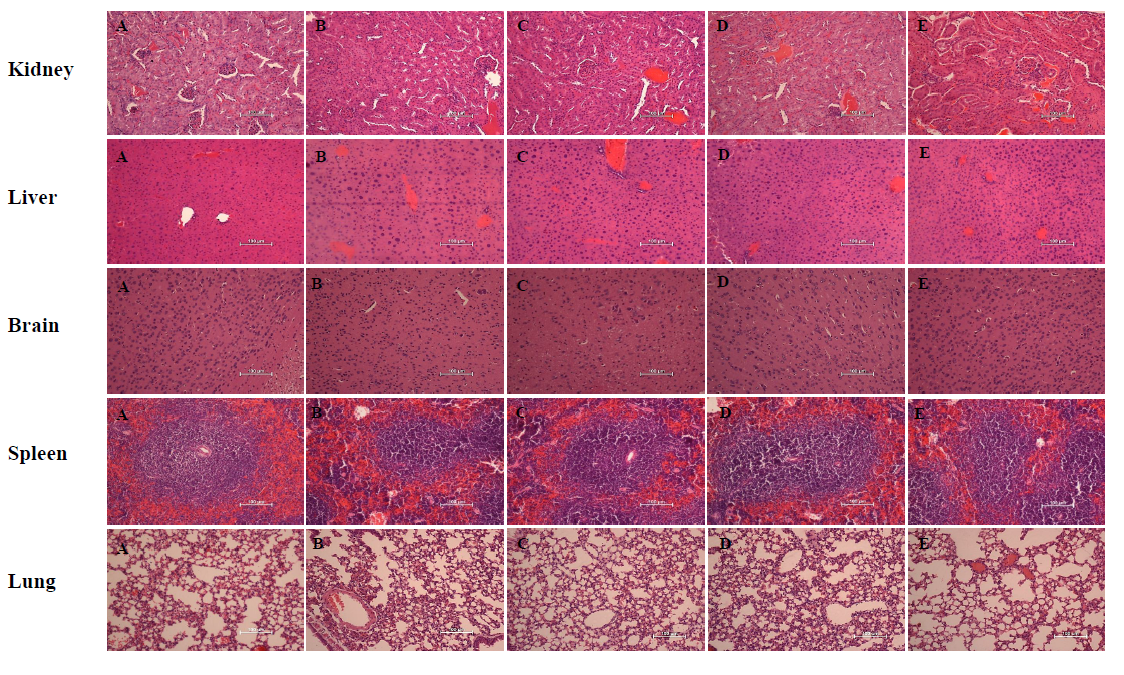

Supplement: Figure S1 — (A) Control group without treatment, (B) mice injected with RN7-IN10 (100 mg/kg). (C) Mice injected with RN7-IN9 (100 mg/kg). Mice injected with RN7-IN8 (100 mg/kg). Mice injected with RN7-IN6 (100 mg/kg). Magnification at 200X, H & E staining. Bar indicates 100 µm. [file peerj-05-3887-s006.png]

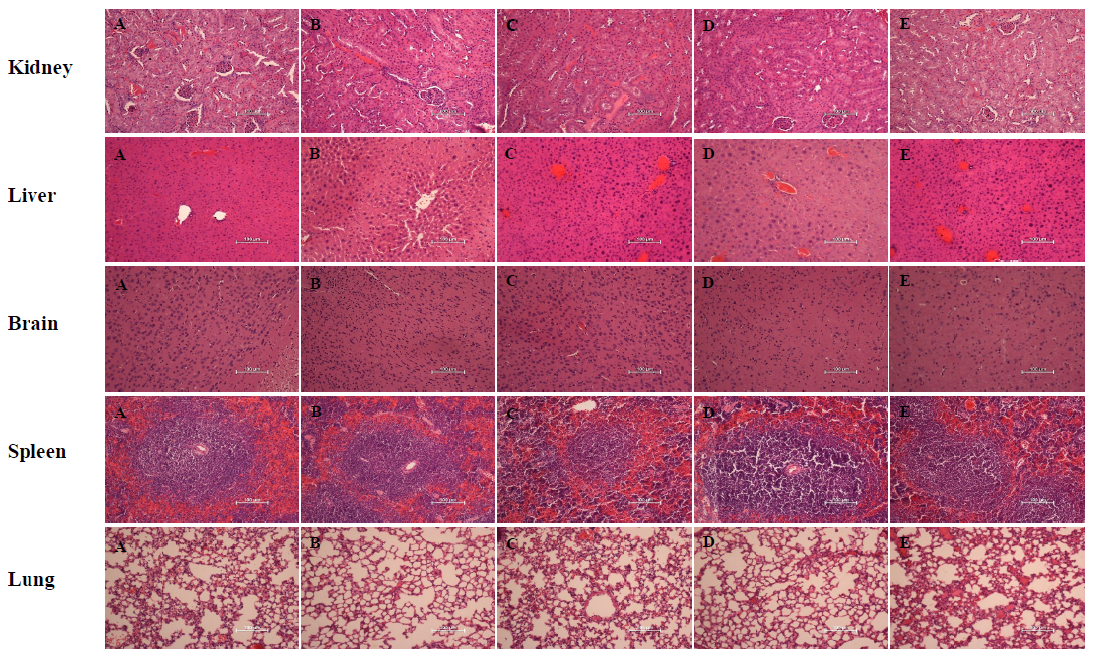

Supplement: Figure S2 — Mice injected with RN7-IN6 (20 mg/kg). Magnification at 200X, H & E staining. Bar indicates 100 µm. [file peerj-05-3887-s007.png]

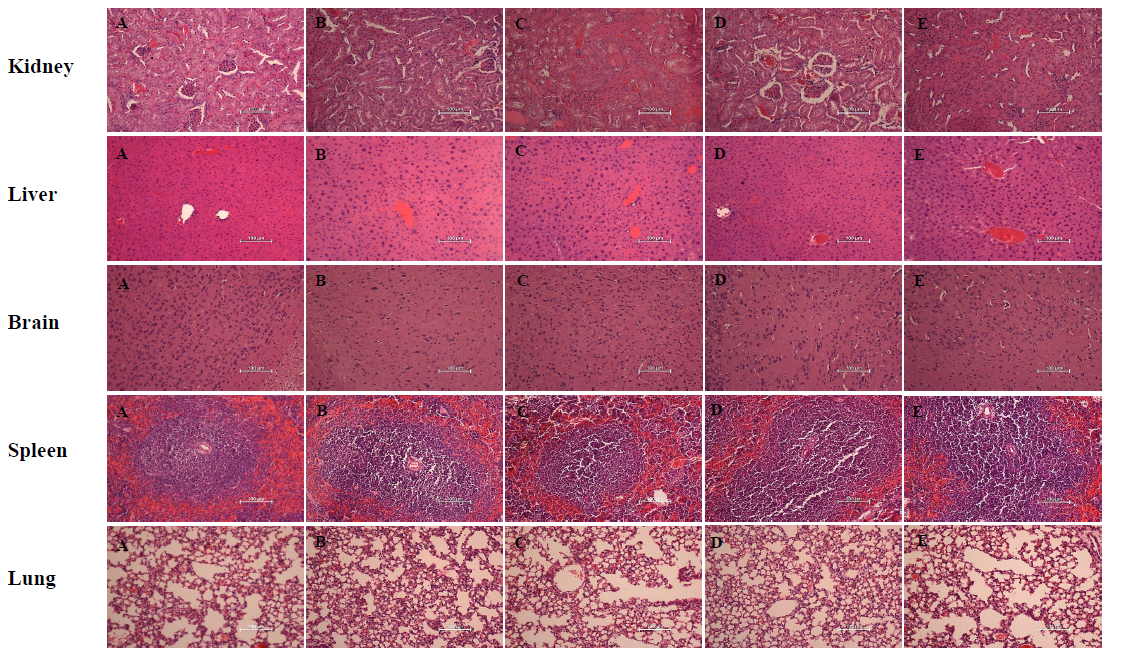

Supplement: Figure S3 — (A) Control group without treatment, (B) mice injected with RN7-IN10 (20 mg/kg). (C) Mice injected with RN7-IN9 (10 mg/kg). Mice injected with RN7-IN8 (20 mg/kg). Mice injected with RN7-IN6 (10 mg/kg). Magnification at 200X, H & E staining. Bar indicates 100 µm. [file peerj-05-3887-s008.png]
