# Supplementary material for: Mechanisms of action and in vivo antibacterial efficacy assessment of five novel hybrid peptides derived from Indolicidin and Ranalexin against Streptococcus pneumoniae
Source: PeerJ. 2017 Oct 5;5:e3887. doi: 10.7717/peerj.3887 (PMC5632533; doi:10.7717/peerj.3887)
Supplement: Table S1 [file peerj-05-3887-s009.docx]

**Table S1.** **Whole blood heamatogram and serum biochemistry of mice treated with four hybrid peptides via SC route.**

| **Parameter** | **SC treatment^a^** | | | | |
| --- | --- | --- | --- | --- | --- |
|  | **Control** | **RN7-IN10 (20 mg/kg)** | **RN7-IN9 (10 mg/kg)** | **RN7-IN8 (20 mg/kg)** | **RN7-IN6 (10mg/kg)** |
| **Whole blood** |  |  |  |  |  |
| Erythrocytes, RBC (10^6^/mm^3^) | 8.31±0.4 | 8.04±1.18 | 9.22±0.98 | 9.17±0.83 | 9.59±0.92 |
| Hemoglobin Hgb (g/dl) | 14.3±0.82 | 13.75±1.77 | 15.75±1.4 | 15.82±1.11 | 16.17±1.44 |
| Mean corpuscular volume, MCV (µm^3^) | 51.5±0.57 | 50±2.16 | 51±1.82 | 50.25±0.95 | 50±1.82 |
| Mean corpuscular haemoglobin concentration, MCHC (g/dl) | 33.55±0.91 | 34.45±1.35 | 34.2±1.37 | 34.38±0.52 | 32.87±0.42 |
| Mean corpuscular haemoglobin, MCH (pg) | 17.02±0.53 | 17.08±0.4 | 17.1±0.53 | 17.32±0.49 | 16.85±0.3 |
| platelet Counts, PLT (10^3^/mm^3^) | 545±98.88 | 568.75±81.73 | 449.75±100.23 | 445.75±47.21 | 273±43.55 |
| Hematocrit, HCT % | 42.67±2.5 | 41.5±3.7 | 46.15±5.19 | 46.15±3.69 | 49.2±4.43 |
| White blood cells, WBC (10^3^/mm^3^) | 4.4±0.52 | 3.8±0.2 | 4.32±0.55 | 3.47±0.48 | 3.86±0.34 |
| Lymphocytes % | 49.75±11.3 | 52.2±3.3 | 46.47±5.48 | 43.85±6.28 | 51.85±3.3 |
| Monocytes % | 2.3±0.6 | 2.2±0.28 | 1.95±0.1 | 1.92±0.15 | 2.8±0.25 |
| Granulocytes % | 66.25±7.6 | 46.6±7.47 | 55.55±4.52 | 58.62±9.82 | 51.12±5.16 |
| Eosinophil % | 2.5±0.6 | 2.82±0.25 | 2.77±0.54 | 2.42±0.61 | 2.7±0.71 |
| **Serum biochemistry** |  |  |  |  |  |
| Aspartate aminotransferase, AST | 173.8±13.1 | 175±25.41 | 188.33±11.22 | 170.75±14.52 | 158.25±18.9 |
| Alanine transaminase, ALT | 43.5±11.45 | 51.25±11.9 | 43.17±13.81 | 50±5.49 | 43.32±10.3 |
| Alkaline phosphatase, ALP | 130.25±6.9 | 89.87±8.07 | 131.25±19.7 | 134±8.24 | 106.56±20.1 |
| Creatinine | 39.6±0.61 | 37.8±3.43 | 35±1.27 | 43.4±0.8 | 34.89±0.25 |
| Urea | 11.13±3.24 | 9.2±0.8 | 8.84±1.04 | 9.61±3.02 | 9.07±2.02 |
| Total bilirubin | 2.1±0.1 | 2.1±0.35 | 2.2±0.13 | 1.84±0.12 | 2.03±0.14 |

^a^ Given for three doses (1hr, 12hr, and 24hr).

Statistical analysis between treatment groups and untreated control group was performed using one-way ANOVA with *post hoc* Dunnett-t test.

Mean value (s) showing significant difference (p ≤ 0.05) as compared to the untreated control was highlighted:

Highlighted in yellow: RN7-IN10 treated mice (granulocytes, p = 0.0172; ALP, p = 0.0037).

Highlighted in blue: RN7-IN6 treated mice (platelet counts, p = 0.048).
