# Supplementary material for: Mechanisms of action and in vivo antibacterial efficacy assessment of five novel hybrid peptides derived from Indolicidin and Ranalexin against Streptococcus pneumoniae
Source: PeerJ. 2017 Oct 5;5:e3887. doi: 10.7717/peerj.3887 (PMC5632533; doi:10.7717/peerj.3887)
Supplement: Table S2 [file peerj-05-3887-s010.docx]

**Table S2.** **Whole blood heamatogram and serum biochemistry of mice treated with four hybrid peptides via IN route.**

| **Parameter** | **IN treatment^a^** | | | | |
| --- | --- | --- | --- | --- | --- |
|  | **Control** | **RN7-IN10 (20 mg/kg)** | **RN7-IN9 (10 mg/kg)** | **RN7-IN8 (20 mg/kg)** | **RN7-IN6 (10mg/kg)** |
| **Whole blood** |  |  |  |  |  |
| Erythrocytes, RBC (10^6^/mm^3^) | 9.42±1.42 | 9.62±0.79 | 9.36±0.9 | 8.95±0.53 | 9.12±0.25 |
| Hemoglobin Hgb (g/dl) | 15.5±2.35 | 15.47±1.07 | 14.9±1.32 | 14.42±0.76 | 15±0.28 |
| Mean corpuscular volume, MCV (µm^3^) | 51.5±0.57 | 50.75±1.7 | 42±6.05 | 50.25±1.25 | 52.66±1.24 |
| Mean corpuscular haemoglobin concentration, MCHC (g/dl) | 32.42±0.43 | 28.3±8.15 | 31.85±0.17 | 32.22±0.4 | 31.7±0.21 |
| Mean corpuscular haemoglobin, MCH (pg) | 16.67±0.27 | 20±7.87 | 15.95±0.33 | 16.12±0.33 | 16.5±0.17 |
| platelet Counts, PLT (10^3^/mm^3^) | 578.5±94.77 | 619.5±100.42 | 656.75±76.9 | 612±130.37 | 597.66±123.23 |
| Hematocrit, HCT % | 48.37±7.83 | 47.95±3.18 | 46.82±4.23 | 44.8±2.9 | 47.43±0.61 |
| White blood cells, WBC (10^3^/mm^3^) | 4.2±0.87 | 5.2±1.44 | 5.2±2.35 | 3.95±0.54 | 3.6±0.35 |
| Lymphocytes % | 54.82±9.23 | 58.5±7.53 | 41.27±10.56 | 45.6±14.31 | 47.67±12.2 |
| Monocytes % | 3±0.43 | 2.2±0.37 | 2.52±0.27 | 3.22±0.68 | 2.7±0.57 |
| Granulocytes % | 57.18±4.95 | 66.6±21.64 | 56.2±10.81 | 52.9±9.54 | 37.67±3.8 |
| Eosinophil % | 2.7±1.05 | 2.7±0.97 | 3.2±1.2 | 4.3±3.6 | 3.5±0.9 |
| **Serum biochemistry** |  |  |  |  |  |
| Aspartate aminotransferase, AST | 174.75±12.6 | 172±8.78 | 141.2±23.08 | 176.3±31.91 | 140±7.2 |
| Alanine transaminase, ALT | 49.9±6.48 | 66.9±17.02 | 41.22±15.75 | 44.5±18.9 | 43.2±7.56 |
| Alkaline phosphatase, ALP | 149.3±2.62 | 133.3±10.1 | 127.75±6.07 | 128.5±21.7 | 138.33±5.5 |
| Creatinine | 40.15±2.7 | 40.94±0.83 | 37.66±1.26 | 40.56±1.74 | 40.15±3.6 |
| Urea | 9.95±1.63 | 9.3±1.68 | 9.6±1.02 | 8.89±1.30 | 10.14±1.52 |
| Total bilirubin | 2.04±0.12 | 2.15±0.14 | 2.01±0.27 | 2.21±0.18 | 1.95±0.08 |

^a^ Given for three doses (1hr, 12hr, and 24hr).

Statistical analysis between treatment groups and untreated control group was performed using one-way ANOVA with *post hoc* Dunnett-t test.

Mean value (s) showing significant difference (p ≤ 0.05) as compared to the untreated control was highlighted:

Highlighted in yellow: RN7-IN9 treated mice (MCV, p = 0.001).

Highlighted in blue: RN7-IN6 treated mice (granulocytes, p = 0.0482).
