# Supplementary material for: Mechanisms of action and in vivo antibacterial efficacy assessment of five novel hybrid peptides derived from Indolicidin and Ranalexin against Streptococcus pneumoniae
Source: PeerJ. 2017 Oct 5;5:e3887. doi: 10.7717/peerj.3887 (PMC5632533; doi:10.7717/peerj.3887)
Supplement: Table S3 [file peerj-05-3887-s011.docx]

**Table S3.** **Whole blood heamatogram and serum biochemistry of mice treated with four hybrid peptides via IP route.**

| **Parameter** | **IP treatment^a^** | | | | |
| --- | --- | --- | --- | --- | --- |
|  | **Control** | **RN7-IN10 (20 mg/kg)** | **RN7-IN9 (10 mg/kg)** | **RN7-IN8 (20 mg/kg)** | **RN7-IN6 (10mg/kg)** |
| **Whole blood** |  |  |  |  |  |
| Erythrocytes, RBC (10^6^/mm^3^) | 8.895±0.86 | 9.16±0.92 | 8.06±1.04 | 8.14±0.34 | 8.71±0.44 |
| Hemoglobin Hgb (g/dl) | 15.5±1.11 | 15.85±1.27 | 13.9±1.67 | 14.55±0.17 | 15.02±0.59 |
| Mean corpuscular volume, MCV (µm^3^) | 52±3.16 | 50.75±2.06 | 51.5±2.64 | 52.75±0.95 | 50.5±1.29 |
| Mean corpuscular haemoglobin concentration, MCHC (g/dl) | 33.33±0.96 | 33.85±0.60 | 33.63±0.34 | 34.17±1.13 | 33.42±0.62 |
| Mean corpuscular haemoglobin, MCH (pg) | 17.28±0.78 | 17.6±0.46 | 17.25±0.78 | 17.92±0.56 | 16.67±1.07 |
| platelet Counts, PLT (10^3^/mm^3^) | 673.75±211.42 | 535±150.11 | 674±118.72 | 472.5±100.52 | 540.75±107.41 |
| Hematocrit, HCT % | 46.2±4.69 | 47.67±5.6 | 41.42±5.25 | 42.73±1.66 | 44.97±1.75 |
| White blood cells, WBC (10^3^/mm^3^) | 5.33±0.46 | 4.2±0.55 | 3.7±1.54 | 4.18±0.36 | 4.1±0.72 |
| Lymphocytes % | 62.12±6.9 | 51.03±16.69 | 43.25±4.07 | 53.7±8.22 | 54.6±3.8 |
| Monocytes % | 2.77±0.47 | 2.8±0.5 | 2.47±0.25 | 2.77±0.88 | 2.53±0.33 |
| Granulocytes % | 56.1±7.02 | 46.27±16.41 | 53.02±4.07 | 53.38±12.35 | 57.87±11.66 |
| Eosinophil % | 3.28±0.71 | 2.23±1.61 | 3.95±2.56 | 2.34±0.63 | 3.22±1.47 |
| **Serum biochemistry** |  |  |  |  |  |
| Aspartate aminotransferase, AST | 168±23.33 | 181.66±38.27 | 172.33±26.35 | 148.9±21.51 | 176±19.31 |
| Alanine transaminase, ALT | 58.37±3.37 | 52.76±12.22 | 51.54±5.83 | 62.1±23.84 | 34.36.4±1.7 |
| Alkaline phosphatase, ALP | 144.5±17.29 | 124.33±10.6 | 89.76±10.7 | 115.4±34.9 | 114.97±24.9 |
| Creatinine | 39.85±2.67 | 38.1±6.97 | 37.3±3.51 | 39.2±3.81 | 41.47±4.2 |
| Urea | 9.86±1.67 | 9.6±2.11 | 10.56±5.2 | 10.34±2.36 | 8.97±1.17 |
| Total bilirubin | 2.05±0.1 | 1.82±0.03 | 1.9±0.02 | 1.98±0.11 | 2.1±0.1 |

^a^ Given for three doses (1hr, 12hr, and 24hr).

Statistical analysis between treatment groups and untreated control group was performed using one-way ANOVA with *post hoc* Dunnett-t test.

Mean value (s) showing significant difference (p ≤ 0.05) as compared to the untreated control was highlighted:

Highlighted in yellow: RN7-IN9 treated mice (lymphocytes, p = 0.0445; ALP, p = 0.0187).

Highlighted in blue: RN7-IN6 treated mice (ALT, p = 0.0425).
